# Analysis of single-nucleotide polymorphisms in genes associated with triple-negative breast cancer

**DOI:** 10.3389/fgene.2022.1071352

**Published:** 2022-12-06

**Authors:** Vigneshwaran G., Qurratulain Annie Hasan, Rahul Kumar, Avinash Eranki

**Affiliations:** ^1^ Department of Biomedical Engineering, Indian Institute of Technology Hyderabad, Hyderabad, Telangana, India; ^2^ Department of Genetics and Molecular Medicine, Kamineni Hospitals, Hyderabad, Telangana, India; ^3^ Department of Biotechnology, Indian Institute of Technology Hyderabad, Hyderabad, Telangana, India

**Keywords:** oncogenomics, computational biology, insilico, single nucleotide polymorphism, triple negative breast cancer

## Abstract

Triple-negative breast cancer (TNBC) is a rare variant of breast cancer (BC) known to be aggressive and refractory. TNBC lacks effective early diagnostic and therapeutic options leading to poorer outcomes. The genomic landscape and alterations leading to BC and TNBC are vast and unclear. Single nucleotide polymorphisms (SNPs) are a widespread form of genetic alterations with a multi-faceted impact on multiple diseases, including BC and TNBC. In this study, we attempted to construct a framework that could identify genes associated with TNBC and screen the SNPs reported in these genes using a set of computational predictors. This framework helped identify *BRCA1, BRCA2, EGFR, PIK3CA, PTEN,* and *TP53* as recurrent genes associated with TNBC. We found 2%–29% of reported SNPs across genes to be typed pathogenic by all the predictors in the framework. We demonstrate that our framework prediction on BC samples identifies 99% of alterations as pathogenic by at least one predictor and 32% as pathogenic by all the predictors. Our framework could be an initial step in developing an early diagnosis of TNBC and potentially help improve the understanding of therapeutic resistance and sensitivity.

## Introduction

TNBC is an aggressive and refractory form of BC and accounts for about 15% of total BC cases (Cleator et al., 2007). The absence of three primary receptors characterises TNBC: estrogen receptor (ER), progesterone receptor (PR), and the human epidermal growth factor 2 receptor (HER2) (Sorlie et al., 2001). Most TNBC tumors are also basal-like subtypes due to their unique genomic profiling and their striking resemblance to basal cells that line the breast ducts in contrast to other subtypes of BC (Thike et al., 2010). TNBC often presents with higher metastases, recurrence, and poor survival rates (Dent et al., 2009). Most conventional therapies used to treat different BC types by targeting ER, PR, or HER2 receptors are ineffective, making TNBC one of the most resistant forms of BC with a poor prognosis (Anders and Carey, 2008).

Personalised cancer diagnosis and therapy could be critical to effective treatment outcomes. TNBC’s incidence, tumorigenesis, progression, and therapeutic response may not be confined to any single causative factor but a consequence of multiple causes, including genetic, ethnic, and lifestyle factors (Jeronimo and Weller, 2017). The degree of oncogenesis and therapeutic sensitivity may vary even between individuals, possibly due to genomic diversity (Voon and Kong, 2011). Understanding the overall tumor genomic landscape can aid in designing and implementing customised and potentially effective therapies (Weitzel et al., 2011).

Tumor-associated alterations can be either germline (hereditary) or somatic (acquired); thus, understanding the underlying characteristics of any tumor is vital in managing the disease (Wu et al., 2019). SNPs are cosmopolitan alterations and are known to have more impact on the underlying condition than other types (Nelson et al., 2004). SNPs that impact the respective amino acid (AA) sequence are known as non-synonymous (nsSNP) or missense variants. The role of these SNPs in a particular disease can be defined by the nature, location, and genotype-phenotype association (Shastry, 2009). Each gene can harbor a humongous number of SNPs, increasing the burden of identifying a candidate SNP. This emphasises the need to converge on a subset of all the possible SNPs, in addition to clinical correlation, which could help us devise a highly confident subset of SNPs on any gene towards any phenotype. Yet analysing all SNPs on a clinical level is a laborious and overarching step. Thus, a way to filter the SNPs is needed to curate potentially pathogenic SNPs.

**FIGURE 1:**
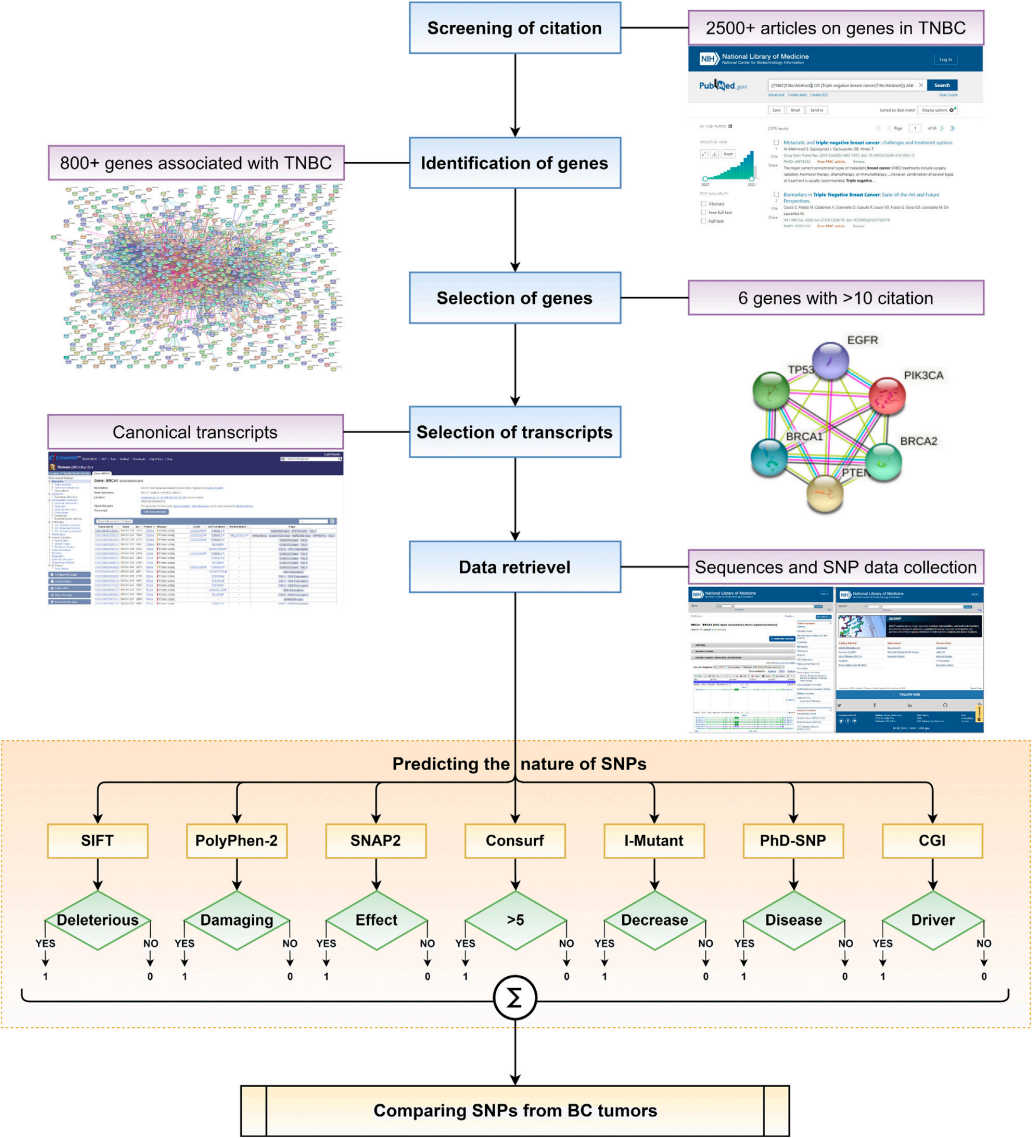
Schematic representation of overall framework in this work to understand the role of various SNPs in disease causing.

Current computational predictors have evolved to analyse and possibly predict the impact of an SNP on a disease (Hasnain et al., 2020). However, there is a lack of a framework of computational predictors to systematically assess SNPs that could have a pathogenic effect. Herein, we utilise a set of selected *in silico* predictors to identify and characterise SNPs associated with recurrent genes in TNBC, having more than ten independent publications supporting their association. The primary objective of the work is to build a framework (Figure 1) to identify SNPs that may be pathogenic and possibly disease-causing. Secondly, to understand the number of predictors required to predict the effect of an SNP in causing TNBC. Finally, we compare the SNPs predicted using the proposed framework against SNPs present in tumors obtained from patients suffering from BC and TNBC.

## Materials and methods

The workflow of the framework used is illustrated in Figure 1. SNPs reported in the selected genes were subjected to computational screening with the help of a framework of predictors curated to cover different aspects of computational functional prediction for an SNP. The predictors used in the framework could provide a systematic route to identify the pathogenicity of an SNP, which is detailed in the subsequent subsections.

### Identifying recurrent genes

Identifying genes associated with TNBC was done with the help of articles indexed on PubMed. A search was performed with keywords of interest, including “TNBC” and “genes”, resulting in about 2540 articles. A preliminary screening of these articles resulted in 800 + genes associated in one or more studies with TNBC in terms of expression or alterations favouring the disease progression. Amongst these, genes associated with TNBC in more than ten independent articles were identified as recurrent genes and were subjected to computational analysis to identify pathogenic SNPs.

### Collection of datasets

Protein and Nucleotide sequences were obtained in FASTA format from the NCBI database. The sequence transcripts were selected with the help of the Ensembl genome browser by identifying canonical transcripts with Ensembl and MANE select flags. NCBI dbSNP database was used to obtain datasets for SNPs reported for a given gene.

### Functional impact prediction

SNPnexus (Oscanoa et al., 2020) and SNAP2 (Hecht et al., 2015) were utilised to predict the pathogenicity of an SNP. SNPnexus is a consortium of multiple predictors to predict a particular SNP’s nature and functional impact. Some of the predictors embedded in SNPnexus include, but not limited to, are SIFT and PolyPhen-2, which predict the effect of a given SNP and their resultant AA alteration based on their respective confidence scores. Few of the SNPs were unannotated by the platforms pertaining to the nature and position of SNPs on the transcript.

SIFT (Vaser et al., 2016) (sorting intolerant from tolerant) uses PSI-BLAST-based multiple sequence alignment (MSA) followed by calculation of diversity with the help of Dirichlet estimation and predicts a tolerance index score to designate an SNP to be “deleterious” or “tolerated”. A tolerance score of ≤0.05 on a scale of 0–1 is termed “deleterious”, while the others are “tolerated”. Confidence in SIFT prediction is based on the number of sequences available for alignment performed by the program. Predictions are labelled “low confidence” when sequences aligned are highly identical, thus increasing false positive rates. To eliminate this, we considered “deleterious—low confidence” SNPs as “tolerated”. All SNPs predicted as “deleterious” were considered pathogenic under the framework.

PolyPhen-2 (Adzhubei et al., 2010) (polymorphism phenotyping) exploits an ML-based probabilistic classifier along with its own pipeline of MSA to predict the functional significance of an SNP with the help of various sequence and structure-based features of the substitution site. Based on the prediction and rate of false positives (Naïve Bayes posterior probability), it types the SNPs as “benign”, “possibly damaging”, or “probably damaging” (decreasing order of false-positive rates). All SNPs predicted as not “benign” were considered pathogenic under the framework.

SNAP2 (Hecht et al., 2015) (screening for non-acceptable polymorphisms) works on an ML-based neural network trained to account for multiple input sequence features, including MSA, secondary structure prediction, and solvent accessibility. It predicts the effect of every possible substitution in a given protein sequence and generates a heatmap along with a numerical scoring on the scale of 100 to -100, with 100 to 1 predicted to have an “effect” on the protein while those between 0 and -100 are “neutral”. All SNPs predicted to have an “effect” were considered pathogenic under the framework.

### Structural impact prediction

SNPs are known to alter the stability of a protein based on their position and the type of alterations they bring in the AA sequence. We utilised I-Mutant2.0 (Capriotti et al., 2005) to predict whether our SNPs “increase” or “decrease” the protein’s stability and strength. I-Mutant2.0 is a support vector machine (SVM) based predictor for the effect of single-site mutations by calculating the Gibbs free energy (DDG) value of the mutated against the native protein. Based on the DDG value, I-Mutant2.0 designates the SNP to “decrease” or “increase” the stability of the native protein with an accuracy of 77% (Capriotti et al., 2005). All SNPs predicted to “decrease” the stability were considered pathogenic under the framework.

### Disease association prediction

PhD-SNP (Capriotti et al., 2006) (predictor of human deleterious single nucleotide polymorphisms) was utilised to predict whether the SNP could have an association with a disease phenotype. It is an SVM-based predictor that utilises sequence information to estimate the disease association of an SNP by a 20-element vector-based conservation index with more than 78% accuracy (Capriotti et al., 2006). It predicts the SNP as “disease” associated when the score is ≤0.5, while the remaining are typed “neutral”. All SNPs predicted to have “disease” association were considered pathogenic under the framework.

### Sequence conservation prediction

ConSurf (Armon et al., 2001) was utilised to predict the evolutionary conservativeness for each position of a given protein sequence. It is a web-based predictor that takes an AA sequence as input and performs MSA as the initial step. Further, it performs phylogenetic tree construction, 2D and 3D structure predictions, and the calculation of position-specific conservation scores with the help of Bayesian and ML algorithms. Each AA in a protein sequence is graded on a scale of 1–9, with 1 - 3 considered variable, 4—6 as moderately conserved, and 7—9 as highly conserved regions. All SNPs predicted to have a score of more than 5 were considered pathogenic under the framework.

### Oncogenicity prediction

CGI (cancer genome interpreter) (Tamborero et al., 2018) was utilised to predict the role of the given SNP as a “driver” or “passenger” in tumorigenesis and determine the SNPs’ specific responsiveness to a given therapy. CGI is built based on established cancer genomic databases and ML-based BoostDM and OncodriveMut algorithms that perform *in silico* saturation mutagenesis to identify driver mutations. All SNPs predicted to be “driver” were considered pathogenic under the framework.

### Scoring of SNPs by the framework

The collected SNPs for any gene in our list were processed through all seven predictors parallelly and were scored based on their pathogenicity prediction. The score can define the level of pathogenicity of an SNP under the framework. A score of one is given to any SNP if it is predicted to be pathogenic by any predictor. This number increases with every predictor predicting this SNP to be pathogenic up to seven. Likewise, when an SNP is not predicted to be pathogenic by any of the predictor a score of zero is given to the SNP. In summary, every SNP analysed in this study could be scored in a range of 0–7. A score of 0 represents the least pathogenic SNP, while a score of 7 represents the most pathogenic SNP based on our prediction framework.

### Correlation with breast cancer database

cBioPortal (Cerami et al., 2012), an online repository of cancer genomics data, was utilised to obtain breast cancer-specific SNPs. A total of 11,632 breast tumor samples from 24 studies were selected (Supplementary Figure S1), and missense SNPs related to the genes of our study were obtained. The data was compared against SNPs identified in this study to understand the correlation between the framework prediction and SNPs found in breast tumors from cBioPortal.

## Results

Utilising our framework, *BRCA1*, *BRCA2*, *EGFR*, *PIK3CA*, *PTEN,* and *TP53* were identified as recurrent genes from a total of over 800 genes associated with TNBC (Supplementary Table S1). The data relating to these genes were obtained from the Ensembl and NCBI database, which includes nucleotide and AA sequences, details of the transcript, and the SNPs reported on those genes (Table 1).

**TABLE 1:** Details of transcripts and SNPs of the recurrent genes identified by our framework.

Genes	No of AA	BPs	Transcript ID	Total SNPs	AA alterations
*BRCA1*	1863	7088	ENST00000357654	35717	2121
*BRCA2*	3418	11954	ENST00000380152	37637	3710
*EGFR*	1210	9905	ENST00000275493	71845	741
*PIK3CA*	1068	9259	ENST00000263967	32291	342
*PTEN*	403	8515	ENST00000371953	41364	314
*TP53*	393	2512	ENST00000269305	9478	609

### Functional impact

SNPs of all the genes, as mentioned in Table 1, were processed through SNPnexus and were assigned scores based on SIFT and PolyPhen-2. SIFT indexing identified 1080 (50.92%) SNPs as “deleterious”, while PolyPhen-2 indexing identified 752 (35.45%) SNPs as “damaging” in *BRCA1*. Similarly, 1631 (43.96%) and 1853 (49.95%) SNPs in *BRCA2*, 416 (56.14%) and 412 (55.60%) SNPs in *EGFR*, 129 (37.72%) and 162 (47.37%) SNPs in *PIK3CA*, 186 (59.24%) and 199 (63.38%) SNPs in *PTEN*, 402 (66.01%) and 418 (68.64%) SNPs in *TP53* were identified to be “deleterious” by SIFT and “damaging” by Polyphen-2, respectively (Supplementary Table S2a).

Simultaneously, SNAP2 predicted the impact of all the possible substitutions on the native AA sequences for every gene in our list. The given SNPs were screened against the data obtained from SNAP2. SNAP2 prediction identified 1130 (53.28%) SNPs in *BRCA1*, 1156 (31.16%) SNPs in *BRCA2*, 374 (50.47%) SNPs in *EGFR*, 121 (35.38%) SNPs in *PIK3CA*, 186 (59.24%) SNPs in *PTEN* and 417 (68.47%) SNPs in *TP53* to have a possible “effect” on the function of the protein (Supplementary Table S2a).

### Impact on stability

I-Mutant 2.0 predicts the effect of a given SNP to either “increase” or “decrease” the structural stability of the protein. I-Mutant predicted that 1754 (82.70%) SNPs in *BRCA1*, 3094 (83.40%) SNPs in *BRCA2*, 668 (90.15%) SNPs in *EGFR*, 294 (85.96%) SNPs in *PIK3CA*, 281 (89.49%) SNPs in *PTEN* and 523 (85.88%) SNPs in *TP53* were disrupting the protein as they “decrease” the stability (Supplementary Table S2a).

### Disease association

PhD-SNP predicts an SNP to be “neutral” or “disease” associated. PhD-SNP predicted 538 (25.37%) SNPs in *BRCA1*, 968 (26.09%) SNPs in *BRCA2*, 301 (40.62%) SNPs in *EGFR*, 128 (37.43%) SNPs in *PIK3CA*, 165 (52.55%) SNPs in *PTEN* and 301 (49.43%) SNPs in *TP53* to have a possible “disease” association (Supplementary Table S2a).

### Sequence conservation

ConSurf annotates each position of AA sequence based on their conservation across species and defines them accordingly from variable (1) to conserved (9). A total of 1025 (48.33%) SNPs in *BRCA1*, 1706 (45.98%) SNPs in *BRCA2*, 442 (59.65%) SNPs in *EGFR*, 178 (52.05%) SNPs in *PIK3CA*, 196 (62.42%) SNPs in *PTEN* and 421 (69.13%) SNPs in *TP53* occurred in conserved sites of their respective protein sequences with a score of more than 5 (Supplementary Table S2a).

### Oncogenicity

CGI is a predictor of the nature of a given SNP to be a “driver” or “passenger” mutation if present in a tumor. A total of 208 (9.81%) SNPs in *BRCA1*, 735 (19.81%) SNPs in *BRCA2*, 414 (55.87%) SNPs in *EGFR*, 210 (61.40%) SNPs in *PIK3CA*, 176 (56.05%) SNPs in *PTEN* and 401 (65.85%) SNPs in *TP53* were predicted to be “driver” mutations (Supplementary Table S2a).

### Framework-based scoring of SNPs

**FIGURE 2:**
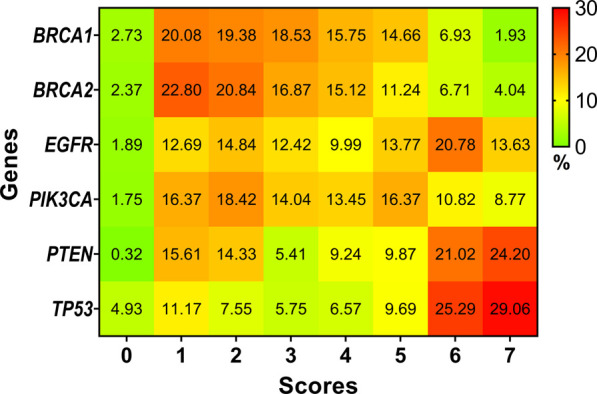
Heat map representing the percentage of SNPs which scored anywhere between a possible score of zero through seven based on our framework.

Different predictors estimate an SNP to be pathogenic or not based on its own algorithm and methodology. All the predictions have been performed and collated to identify SNPs that can be pathogenic with high confidence. We classified the confidence as a proportion to the number of times a particular SNP was typed pathogenic across the prediction. The greater the frequency of a specific SNP to be typed pathogenic, the more confidence the prediction gets. We found 41 (1.93%) SNPs in *BRCA1*, 150 (4.04%) SNPs in *BRCA2*, 101 (13.63%) SNPs in *EGFR*, 30 (8.77%) SNPs in *PIK3CA*, 76 (24.20%) SNPs in *PTEN* and 177 (29.06%) SNPs in *TP53* to have scored 7. In other words, these SNPs were typed pathogenic by all the seven predictors and can be treated as pathogenic SNPs with high confidence. Detailed scoring of all the SNPs screened in the framework is shown in Supplementary Table S2b. Figure 2 explains the distribution of SNPs typed pathogenic by the respective number of predictors.

### Comparing prediction framework over patient-derived SNPs

**FIGURE 3:**
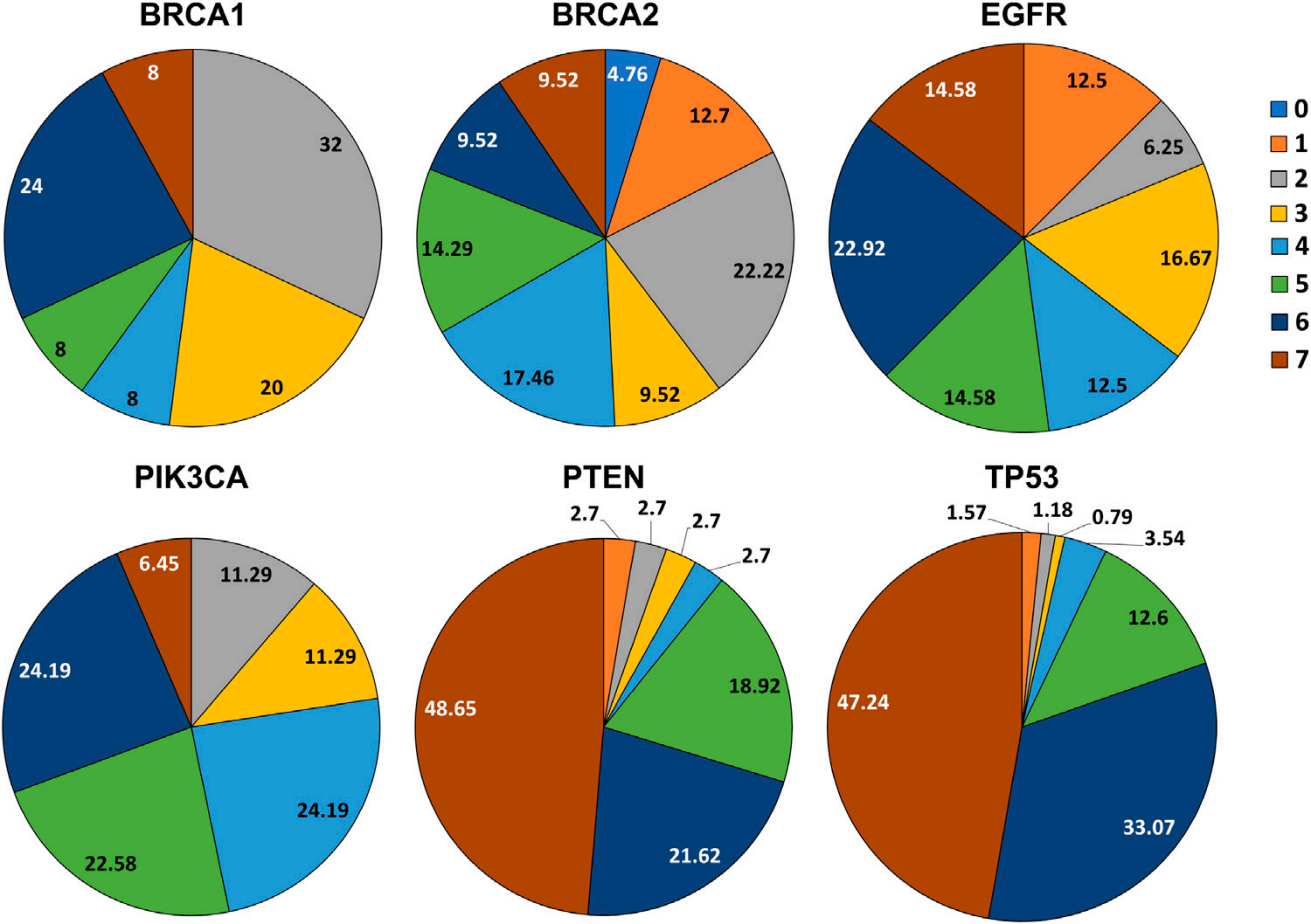
Pie charts representing the percentage of SNPs scored by the framework which overlapped with the alterations derived from patient suffering from breast cancer and TNBC.

We obtained SNPs from the data of breast cancer studies in cBioPortal’s database. Comparing SNPs obtained using the dbSNP dataset scored by our prediction framework with SNPs from patient tumor data (Table 2 and Supplementary Table S2c), we found that 2 (8.0%) SNPs in *BRCA1*, 6 (9.5%) SNPs in *BRCA2*, 7 (14.5%) SNPs in *EGFR*, 4 (6.45%) SNPs in *PIK3CA*, 18 (48.6%) SNPs in *PTEN* and 120 (47.24%) SNPs in *TP53*, have scored 7 in our prediction and were found in breast tumors (Figure 3).

**TABLE 2:** Number of SNPs that were scored in the prediction framework and matched with SNPs reported from breast cancer patients in the cBioPortal.

Genes	No of amino acid alterations		Scores based on prediction framework							
	Patient-derived	Overlapping	0	≤1	≤2	≤3	≤4	≤5	≤6	≤7
*BRCA1*	89	25	0	0	8	13	15	17	23	25
*BRCA2*	151	63	3	11	25	31	42	51	57	63
*EGFR*	102	48	0	6	9	17	23	30	41	48
*PIK3CA*	167	62	0	0	7	14	29	43	58	62
*PTEN*	105	37	0	1	2	3	4	11	19	37
*TP53*	341	254	0	4	7	9	18	50	134	254

## Discussion

The role of SNPs in the initiation and progression of TNBC has not been well established. Herein, we explore the effect of pathogenic SNPs on genes that have a recurrent clinical association with TNBC. Extensive screening of published data identified more than 800 genes associated with TNBC pathogenesis. We curated six recurrently reported genes in TNBC tumors, namely *BRCA1, BRCA2, EGFR, PIK3CA, PTEN and TP53*, identified by our framework. COSMIC cancer gene census has documented all six genes involved as “Tier 1” genes with oncogenic outcomes (Sondka et al., 2018). While each gene may have its own functional capability, they all have some common roles, including cell growth, development, and maintenance (Davis et al., 2014). In TNBC, the functions of the genes are exploited to nurture tumor microenvironment and heterogeneity. BRCAness, or the trait of harbouring BRCA1/2 mutation, is deemed to be a hallmark for screening of BC or TNBC (Kosaka et al., 2020). Some studies have shown that in TNBCs without BRCA1/2 mutations, TP53 seems commonly mutated, while the joint loss of p53 and BRCA1/2 activity could lead to poorer overall survival outcomes (Kim et al., 2016). Analysis of co-expression of EGFR with p53 showed that patients with inverse relationship (EGFR-/p53 + or *vice versa*) had a significantly higher risk of relapse than those with bi-positives or negatives (EGFR-/p53-and EGFR+/p53+) (Levva et al., 2017). Incidence of alterations in PIK3CA and its associated pathways (PI3K/PTEN-axis) is also frequent in TNBC, which serves as a prominent biomarker and renders poor overall outcomes in the clinic (Cossu-Rocca et al., 2015; Philipovskiy et al., 2020). Likewise, PTEN has been reported as a significant negative regulator of pathways related to TNBC and control tumorigenesis (Dey et al., 2013). Genes identified by the framework have interlinked functionality and a crucial role in TNBC tumorigenesis, suggesting the importance of any SNP in these genes in disease progression and refractory behaviour.

**FIGURE 4:**
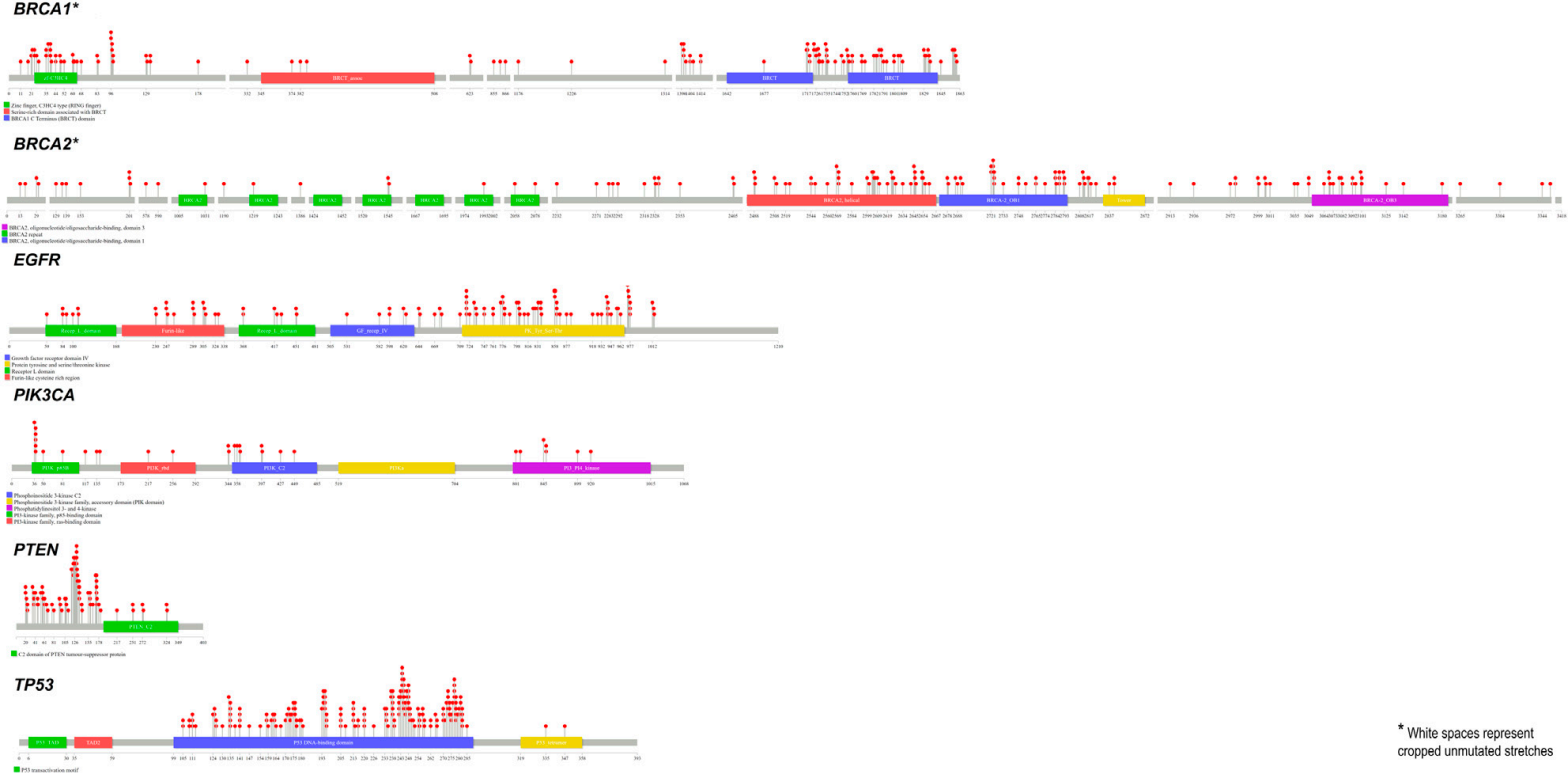
Lollipop plot for the alterations that were typed pathogenic by all the predictors.

SNPs tend to have a significant share among all the alterations found in humans, of which nsSNPs are known to impact the structural stability and functioning of proteins (Yates and Sternberg, 2013). Pertaining to our interest in understanding the impact of alteration on protein functioning, we excluded the synonymous, non-coding and intronic alterations. Further indels, when compared against nsSNPs, seem to have lesser significance in being causatives of complex disorders (Gagliano et al., 2019). Thus, the current study focused on understanding the impact of nsSNPs alone. While each gene can harbour a substantial number of SNPs, analysing all those SNPs functionally and clinically could be an exhaustive approach in understanding the consequences of these SNPs. Further, this task is laborious and likely expensive. In addition, not all SNPs are disease-causing but tend to have a spectrum of consequences based on the position and nature of the substitution. These include the type of AA substitution, domain of the protein, and conservation of the position across species, amongst others (Shastry, 2009). Using computational methodology to filter out pathogenic SNPs could help us identify a subset of pathogenic and potentially disease-causing SNPs with high confidence. We tried identifying nsSNPs reported in these six genes and utilised the framework of predictors with complementary functionalities. The analysis of more than 300 SNPs per gene identified 4%–28% of the SNPs to be predicted pathogenic by all the predictors. We also observed from a lollipop plot (Jay and Brouwer, 2016) of all the SNPs that scored 7 (predicted pathogenic by all predictors) that most of them were found in the functional domains of the respective proteins (Figure 4), suggesting that an SNP in the functional domain of a protein could have a higher impact than other regions in the protein.

The predictors used in our framework were explicitly curated to predict the structural and functional impact of an SNP by determining its conservativeness, impact on structural stability, and role in tumorigenesis, amongst others. SIFT, PolyPhen and SNAP2 were used to predict the functional effect of an SNP by analysing its sequence homology, secondary structures and MSA. The effect and consequence of the structural impact of an SNP were predicted using I-Mutant and PhD-SNP. Phylogenetic conservation prediction and scoring were performed with ConSurf. While CGI was used to predict an SNP’s nature as a driver or passenger mutation in tumorigenesis. The predictors were selected to perform the key predictions necessary for the framework and the ability to handle large data sets. All the predictors involved have been used widely in several literatures on bioinformatics and are considered benchmarks (Hussain et al., 2012; Mustafa et al., 2020; Arshad et al., 2021; Falahi et al., 2021; Poon, 2021). In a simplistic approach, we utilized web servers and package tools to optimise the computational needs; the same framework can be replicated with the help of command line operators of the respective tools.

**FIGURE 5:**
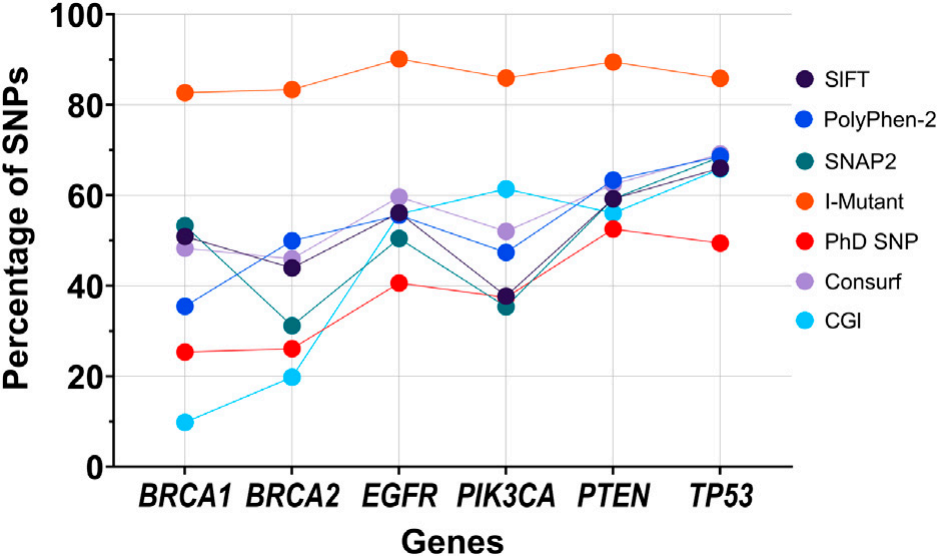
Graph representing the percentage of SNPs typed pathogenic by the each of the seven predictors present in our framework.

Among the predictors used in this study, some predict lesser SNPs to be pathogenic for certain genes when compared to other predictors. This difference is also observed across all six genes (Figure 5). We noticed that I-Mutant predicted most SNPs (more than 80% of SNPs per gene) to decrease the stability of the protein. This indicates that most missense SNPs tend to influence the protein’s structural stability. Amongst all the genes, TP53 was found consistently high in the percentage of SNPs predicted to be pathogenic by any predictor, suggesting that an SNP in TP53 tends to influence many diseases, including BC and TNBC. The trend may also pertain to the varying number of SNPs per gene considered in this study, which might favour disease-causing SNPs being reported while the benign (neutral) SNPs left under-reported might lead to some bias. It can be stressed that the prediction rate can be significantly affected if all the SNPs of a particular gene are considered. For instance, in this study, we obtained results from SNAP2, which grades all possible alterations in an AA sequence. The percentage of alterations that had an “effect” were found to substantially vary from the values obtained when screening for reported SNPs. BRCA2 observed a 12% increase of SNPs to have an “effect” by SNAP2, while TP53 reported a 12% decrease. The decrease in the percentage of SNPs typed to have an “effect” suggests an increased focus of research on TP53 when compared to the other genes involved, or the presence of TP53 SNPs in multiple tumor types (Guimaraes and Hainaut, 2002), leading to the increase in the number of pathogenic SNPs being reported on the gene. This further suggests that the number of predictors required to analyse a gene can vary, suggesting a potential weighted analysis-based approach to normalise the framework to work irrespective of the gene under consideration.

The comparison between results from the prediction framework and SNPs found in breast tumors obtained from the online repository further validates the prediction. We could observe 6%–47% of our predicted pathogenic SNPs (score 7) present in patients diagnosed with breast cancer. Overall, we identified 99.37% (486) of the overlapping SNPs (489) to be scored at least 1 or above in our prediction, suggesting solid confidence in our framework. Similarly, we identified 73.2% (358) of the overlapping SNPs to have scored more than 4, while 32.1% (157) scored exactly 7 by the prediction framework. In addition, we also identified 9 breast tumor samples diagnosed as TNBC from the cBioPortal dataset (BC specific). Out of the 9 samples, 6 (5 patients) harboured SNPs in the genes involved in this study. Two SNPs, *R273C* and *Y163D* in *TP53,* scored 7 and 6 by our prediction, respectively. An SNP in *PIK3CA, H1047R,* which was found in two patients, was scored 5 by our prediction. A *BRCA2* SNP *K3267R* was scored 1 (by ConSurf), stating its conservation. This study did not analyse other SNPs, including *BRCA1—R1085I, PTEN—F206L* and *TP53—R175H*. These results support the *in-silico* prediction framework’s clinical correlation, thus proposing a possible route to identify pathogenic SNPs leading to BC or TNBC. In addition, we analysed the occurrence of the SNPs in all the patient-derived breast tumor samples (Supplementary Table S2d). Since a significant fraction of the SNPs were reported only once or twice on the entire cohort, we defined a 90-percentile cut-off to highlight SNPs reported in multiple samples with their respective scores from our framework (Table 3).

**TABLE 3:** SNPs that were scored by the framework and reported in ≥90th percentile of breast cancer samples.

Genes	Scores received by the SNPs in our prediction framework							
	0	1	2	3	4	5	6	7
*BRCA1*	*-*	*-*	*R496C, D366N*	*E761K, S915C*	*Y1127H, R979C*	*-*	*C61G*	*-*
*BRCA2*	*N319S*	*-*	*E1493Q, E2175Q*	*A3029V, S3389F, R118C*	*R2268K, P2381S, S1817C*	*E1581Q, S2963L, P2798R*	*Y3049C, E3342K, D1033H*	*Y3049S, D3095G, G2793V*
*EGFR*	*-*	*-*	*-*	*E282K, V592I*	*R1068Q*	*S511Y, E114K*	*E257K, R999H, R671C, R222H*	*R836C, R669Q*
*PIK3CA*	*-*	*-*	*E726K*	*H1047L*	*G1049R, Q546K, M1043I, E453K, C420R*	*E545A, G118D, Q546R, E542K, H1047R*	*Q546P, K111E, N345K, E545K*	*E81K*
*PTEN*	*-*	*-*	*-*	*-*	*-*	*K128N*	*D24N, H93R*	*H61R, R130G, A126V, I135K, C136R, R130P, R130Q*
*TP53*	*-*	*-*	*-*	*-*	*-*	*R337C, H193Y, M237I, V272M*	*S241C, E286K, V173M, V216M, S241F, R280K, K132N, Y236C, Y163C, H179R, H193R, R273H*	*L111P, N239D, C176Y, Y234C, R273L, C238Y, G266E, R280T, G245D, C176F, C141Y, G245S, L194R, R282W, I195T, E285K, Y220C, R273C, R248W, R248Q*

Amino acid changes.

The identified SNPs can be associated with a wide spectrum of disorders caused by the impaired functioning of these genes (Shastry, 2007). Yet the association we found between these genes with TNBC deems critical in correlating these SNPs with TNBC or BC tumorigenesis. Although computational analysis can be rapid and economical, the clinical outcome cannot be established without further functional validation through *in vivo* and *in vitro* studies. The predictors in our framework are a subset of all the *in-silico* predictors available. Expanding the number of predictors involved could assist in better characterisation of the pathogenicity of an SNP. Future work would be directed towards identifying SNPs at sensitive locations such as post-translational modification sites or ligand binding sites and predicting the 3D structural impact of the protein through modelling and molecular dynamic simulations.

The increase in oncological SNPs demands a rapid and cost-effective way of estimating the effect of these SNPs. The framework utilised in this study suggests a possible toolset that can help evaluate the impact of SNPs. The methodology and results obtained can be the initial step in understanding the interplay of SNPs in TNBC, which can further be stepped up to analyse any disease with a genetic background. In addition, this framework could guide the administration of personalised therapies (Supplementary Table S3) that can be used to better treat patients suffering from TNBC tumors housing specific SNPs. TNBC tumors tend to resist conventional generalised treatments and use personalised therapies, including checkpoint blockade (Kwa and Adams, 2018), targeted gene therapies (Kuo et al., 2017) or even others such as radiation (Moran, 2015), radiofrequency ablation (McArthur et al., 2018), cryoablation (Chandra et al., 2016) or high intensity focused ultrasound (Deckers et al., 2015; Eranki et al., 2017; Eranki et al., 2020; Sheybani et al., 2020) fetch better results and improved patient outcomes alone or in combination. When combined with therapeutic options, a framework such as the one proposed in this work on TNBC could lead to enhanced personalised therapies and potentially improved survival outcomes.

## Conclusion

Our study analysed 2121, 3710, 741, 342, 314, and 609 SNPs of *BRCA1*, *BRCA2*, *EGFR*, *PIK3CA*, *PTEN,* and *TP53,* respectively. *In silico* predictors used to analyse these SNPs identified 2%–29% of the SNPs across genes to be identified as pathogenic by all the predictors involved in our framework. The *in-silico* predictors suggest that all these SNPs potentially impact protein function and stability. The SNPs and their resultant AA alterations are suggested to affect tumorigenesis through different pathophysiological pathways significantly. The framework proposed and evaluated herein could help predict SNP’s that may lead to BC or TNBC and complement other currently available predictive markers and therapies.

## Data Availability

The original contributions presented in the study are included in the article/Supplementary Material, further inquiries can be directed to the corresponding author.
