# Supplementary figures and images for: Analysis of single-nucleotide polymorphisms in genes associated with triple-negative breast cancer
Source: Front Genet. 2022 Dec 6;13:1071352. doi: 10.3389/fgene.2022.1071352 (PMC9763624; doi:10.3389/fgene.2022.1071352)

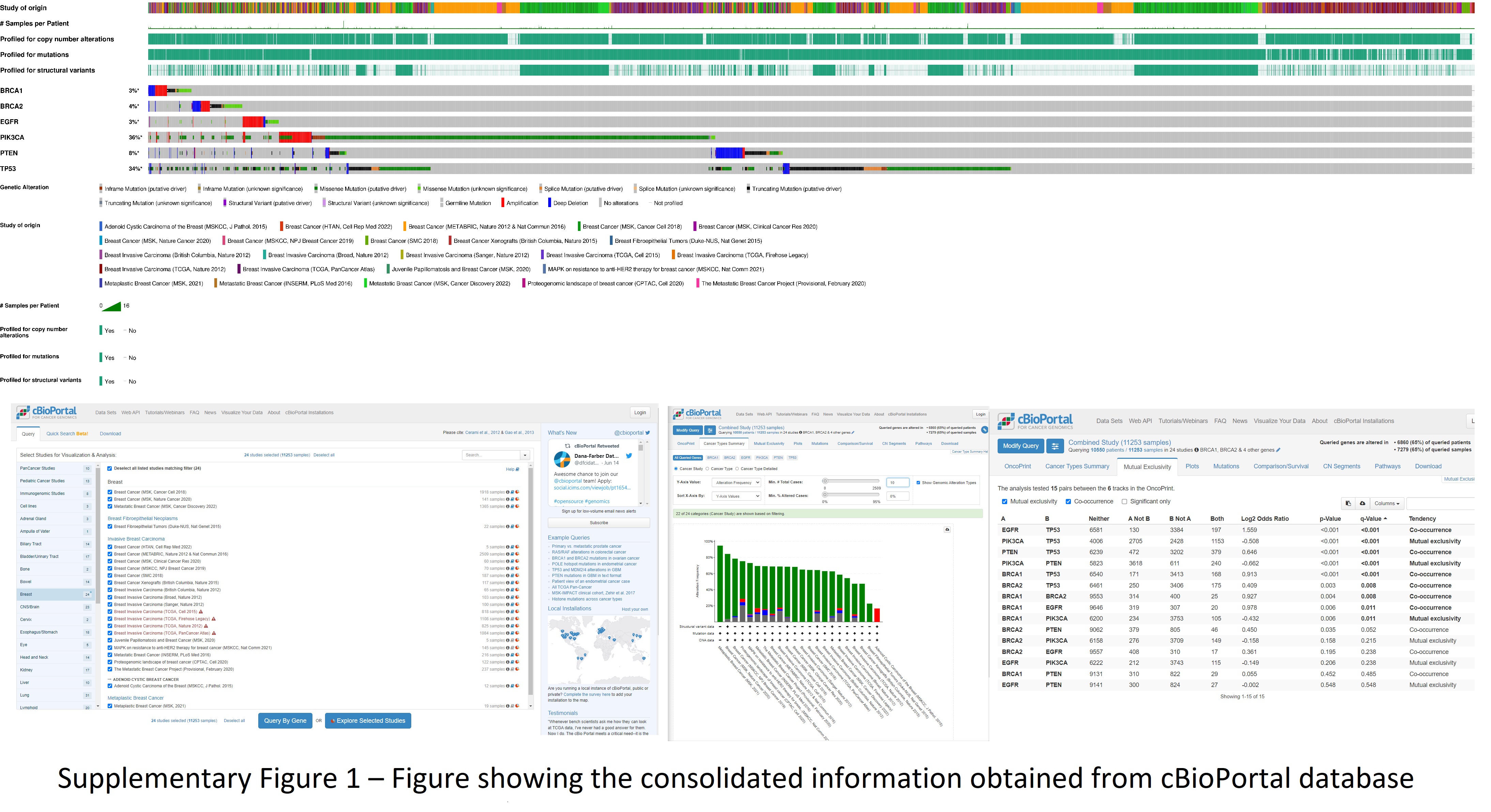

Supplement: Supplementary file 3 [file Image1.JPEG]
